# Transcatheter Aortic Valve Implantation in Nonagenarians: A Comparative Analysis of Baseline Characteristics and 1-Year Outcomes

**DOI:** 10.3390/jcdd12090327

**Published:** 2025-08-26

**Authors:** Murat Can Güney, Engin Bozkurt

**Affiliations:** 1 Department of Cardiology, Faculty of Medicine, Medicana International Ankara Hospital, Atılım University, Söğütözü, 2176. Sk. No. 3, Çankaya 06510, Turkey; 2 Department of Cardiology, Medicana International Ankara Hospital, Söğütözü, 2176. Sk. No. 3, Çankaya 06510, Turkey; drebozkurt@yahoo.com.tr

**Keywords:** transcatheter aortic valve implantation (TAVI), nonagenarians, geriatric cardiology

## Abstract

Background: Transcatheter aortic valve implantation (TAVI) is increasingly used in elderly patients with severe aortic stenosis, yet data on nonagenarians remain limited. This study aimed to compare clinical characteristics and outcomes of patients aged ≥90 years with those aged <90 years undergoing TAVI. Methods: We retrospectively analyzed 620 patients who underwent transfemoral TAVI. Patients were divided into two groups: <90 years (*n* = 545) and ≥90 years (*n* = 75). Baseline clinical, procedural, and outcome data were compared. Results: Nonagenarians had lower body mass index (BMI) and a lower prevalence of comorbidities such as diabetes, hyperlipidemia, and prior coronary artery bypass grafting CABG (all *p* < 0.05). All-cause mortality was higher in nonagenarians at 1 month (8.0% vs. 5.5%, *p* = 0.425), 6 months (9.3% vs. 7.9%, *p* = 0.838), and 1 year (21.3% vs. 16.7%, *p* = 0.405), though these differences were not statistically significant. In-hospital stroke occurred more frequently in patients ≥ 90 years (6.7% vs. 2.2%, *p* = 0.044). Conclusions: Despite a higher rate of in-hospital stroke, nonagenarians undergoing TAVI had comparable mortality outcomes to younger patients. These findings support the feasibility of TAVI in selected very elderly patients, while highlighting the need for tailored stroke prevention strategies. Trial Registration: The trial is retrospectively registered, and a clinical trial number is not applicable.

## 1. Introduction

Projections indicate that by 2050, the world will witness a significant increase in its elderly population, with nonagenarians constituting nearly 5% of the total population [1,2]. This demographic shift results in a growing cohort of patients suffering from severe aortic stenosis (AS) as its prevalence increases exponentially with age [3]. Traditionally, surgical aortic valve replacement (SAVR) has been the definitive treatment for severe AS. However, the associated morbidity and mortality in advanced age often render elderly patients unsuitable candidates for surgery as the perioperative mortality rate for SAVR is approximately 10% in patients aged 90 years and older [4]. Over the past years, transcatheter aortic valve implantation (TAVI) has emerged as a less invasive alternative, offering improved survival and enhanced quality of life for patients deemed inoperable or at high surgical risk [5].

Despite the increasing adoption of TAVI, its efficacy and safety in nonagenarians remain underexplored, as this age group has been underrepresented in major clinical trials [6]. Additionally, the impact of advanced age on the clinical outcomes of TAVI patients remains inconsistent, as observed in various registry analyses and population studies conducted in Europe and the United States [7]. For that reason, advanced age plays a significant role in heart team discussions, and very elderly patients are often considered inoperable or high risk for surgery. This study aims to bridge this knowledge gap by conducting a comparative analysis of baseline characteristics and follow-up outcomes between patients younger than 90 and those aged 90 and above who have undergone the TAVI procedure.

## 2. Methods

This retrospective cohort study analyzed 620 patients who underwent TAVI for severe AS [aortic valve area (AVA) < 1.0 cm^2^ or mean gradient ≥ 40 mm Hg or maximum jet velocity ≥ 4.0 m/s, AVA index < 0.6 cm^2^/m^2^] between July 2011 and December 2024. All participants exhibited symptomatic AS, classified as New York Heart Association (NYHA) functional class II to IV. The institutional ethics committee approved the study (Approval Date: March 2011; Reference No.: 068), and all patients provided informed consent for the use of their clinical data in research, in accordance with institutional and ethical guidelines.

## 3. Patient Evaluation, Selection and Data Collection

Initial assessment of AS severity was conducted using transthoracic echocardiography, followed by either transesophageal echocardiography or electrocardiogram-gated multi-slice computed tomography (MSCT) for further evaluation. A multidisciplinary heart team consisting of experienced clinical and interventional cardiologists, cardiac imaging specialists, cardiovascular surgeons and anesthesiologists determined patient eligibility for TAVI, ensuring a comprehensive evaluation of each case. The decision to proceed with TAVI in patients aged ≥90 years was not based solely on chronological age but on a comprehensive assessment of frailty, functional status, comorbidity burden, and patient preference. Prior to the procedure, all patients underwent invasive coronary angiography to identify any concomitant coronary artery disease (CAD). Presence of >50% stenosis in left main coronary artery or >70% in the epicardial coronary arteries (diameter > 1.5 mm) is defined as obstructive CAD. The initial demographic, laboratory, and echocardiographic information, along with procedural and outcome data, were gathered retrospectively. Patient data was retrieved from the institutions’ electronic databases and individual patient records. Mortality data was collected from the national health service database or through telephone follow-ups. The patient cohort is categorized into two groups based on age at the time of the procedure: those under 90 years and those 90 years or older.

## 4. Procedural Details

Patient eligibility for TAVI was determined by a multidisciplinary heart team. To ensure consistency and reduce variability in our data, only patients who underwent the procedure via the transfemoral access route were included in this analysis. Pre-procedural MSCT was utilized to assess the iliofemoral arteries’ anatomy, focusing on vessel size, calcification, and tortuosity, to confirm suitability for transfemoral access. Five types of aortic valve prostheses were employed: Myval transcatheter heart valve (Meril Life Sciences, Vapi, India), Edwards SAPIEN XT^®^, SAPIEN 3^®^ (Edwards Lifesciences, Irvine, CA, USA), Evolut™ R (Medtronic, Minneapolis, MN, USA) and ACURATE Neo™ (Boston Scientific, Marlborough, MA, USA). Temporary ventricular rapid pacing was induced during the procedure through a pacing lead in the right ventricle. Hemostasis was achieved using percutaneous closure devices, specifically the Prostar XL or Perclose ProGlide 6Fr suture devices, both from Abbott Vascular. Following sheath removal, peripheral angiography assessed the patency of the access site. In transfemoral procedures unsuitable for percutaneous closure, such as those with anterior calcification, surgical cut-down was performed. In technically challenging cases, a safety wire was exchanged in the iliac or femoral artery, or micropuncture needles were utilized to identify the optimal site. Post-successful TAVI procedures, patients were prescribed dual antiplatelet therapy with clopidogrel 75 mg and aspirin 100 mg for a duration of 3 to 6 months. In accordance with evolving guideline recommendations favoring single antiplatelet therapy (SAPT) over dual antiplatelet therapy (DAPT) to reduce bleeding risk, patients treated from 2020 onward were predominantly managed with SAPT—typically aspirin alone—unless specific clinical indications justified DAPT [5]. For patients with atrial fibrillation, antiplatelet treatment was individualized, taking into account their bleeding risk.

## 5. Outcome Measures and Follow-Up

Outcomes were assessed based on the Valve Academic Research Consortium 2 (VARC-2) criteria, which included in-hospital stroke, major vascular and bleeding complications, new pacemaker implantations, the presence and severity of paravalvular leakage (PVL), and device success. Device success was defined as the absence of 30-day mortality, correct positioning of a single prosthesis, and satisfactory prosthesis performance [8]. Ischemic stroke was characterized by the abrupt onset of neurological symptoms or signs that match a specific vascular territory in the brain, spinal cord, or retina within the postoperative 72 h [9]. All suspected events were evaluated by neurology and required neuroimaging confirmation: non-contrast head CT in all cases and diffusion-weighted MRI when clinically feasible; events were captured within 72 h post-procedure. Symptomatic improvement was evaluated using the NYHA classification, with assessments made at 30 days of follow-up. Clinical follow-up was conducted at 30 days, 6 months and one year, with patient status verified through the latest clinical evaluations or telephone interviews.

## 6. Statistical Analysis

The distribution of continuous numerical variables was assessed for normality using the Kolmogorov–Smirnov test, while the assumption of homogeneity of variances was evaluated with Levene’s test. Descriptive statistics were expressed as mean ± standard deviation or median (25th percentile–75th percentile) for continuous numerical variables, whereas categorical variables were presented as frequencies and percentages (%). For continuous numerical variables that met the assumptions of parametric test statistics, differences between age groups were analyzed using Student’s *t*-test. In cases where these assumptions were not met, the Mann–Whitney U test was employed. Unless stated otherwise, Pearson’s χ^2^ test was used for the analysis of categorical variables. In 2 × 2 contingency tables, if at least 1/4 of the expected frequencies were below 5, Fisher’s exact test was applied. If the expected frequencies ranged between 5 and 25, the continuity-corrected χ^2^ test was used. In R × C contingency tables (where at least one categorical variable had more than two possible outcomes), the Fisher–Freeman–Halton test was applied if at least 1/4 of the expected frequencies were below 5. All statistical analyses were conducted using IBM SPSS Statistics version 25 (IBM Corporation, Armonk, NY, USA). A *p*-value of <0.05 was considered statistically significant.

## 7. Results

A total of 620 patients who underwent TAVI were included in the study. Among them, 75 patients (12.1%) were aged 90 years or older, with the oldest being 97 years old. The overall median age of the study population was 79.0 years (interquartile range: 73.3–84.0). Table 1 presents the comparison of baseline characteristics between the age groups. Compared to the group aged <90 years, patients aged ≥90 years had significantly lower body mass index (BMI), history of diabetes mellitus (DM), history of hyperlipidemia (HL), history of coronary artery bypass grafting (CABG), prevalence of bicuspid aortic valve, and baseline glomerular filtration rate (GFR) (*p* < 0.05). No statistically significant differences were observed between the groups for the other evaluated baseline characteristics (*p* > 0.05).

Table 2 shows the comparison of baseline echocardiographic findings between the age groups. In the ≥90 years group, baseline ejection fraction (EF) and septal thickness were significantly higher compared to the <90 years group (*p* = 0.048 and *p* = 0.012, respectively). Conversely, baseline left ventricular end-diastolic diameter (LVEDD) and left ventricular end-systolic diameter (LVESD) were significantly lower in the ≥90 years group (*p* < 0.001). No significant differences were observed in the other echocardiographic parameters between the groups (*p* > 0.05).

Table 3 presents the comparison of procedural characteristics between the age groups. Apart from valve size, no significant differences were detected in procedural characteristics between the groups (*p* > 0.05). However, a significant difference was observed in valve size (*p* = 0.026), primarily driven by the lower frequency of 29 mm valve usage in the ≥90 years group compared to the <90 years group (*p* = 0.012).

**Figure 1:**
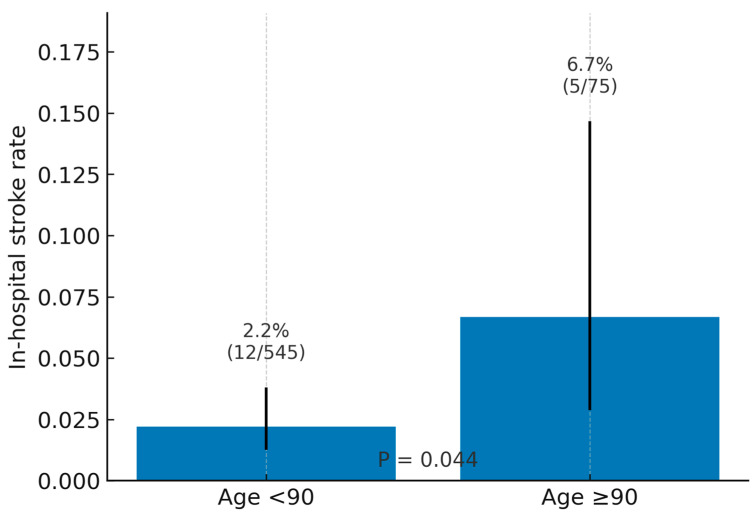
In-hospital stroke rates (with 95% Wilson CIs): bar chart displaying rates for <90 vs. ≥90 (with counts) and the between-group *p* = 0.044 annotation.

Table 4 compares the in-hospital outcomes between the age groups. No significant differences were found in in-hospital outcomes between the groups, except for in-hospital stroke. Patients aged ≥90 years had a significantly higher incidence of in-hospital stroke compared to those <90 years (*p* = 0.044) (Figure 1). Other in-hospital outcomes showed no significant differences between the groups (*p* > 0.05).

**Figure 2:**
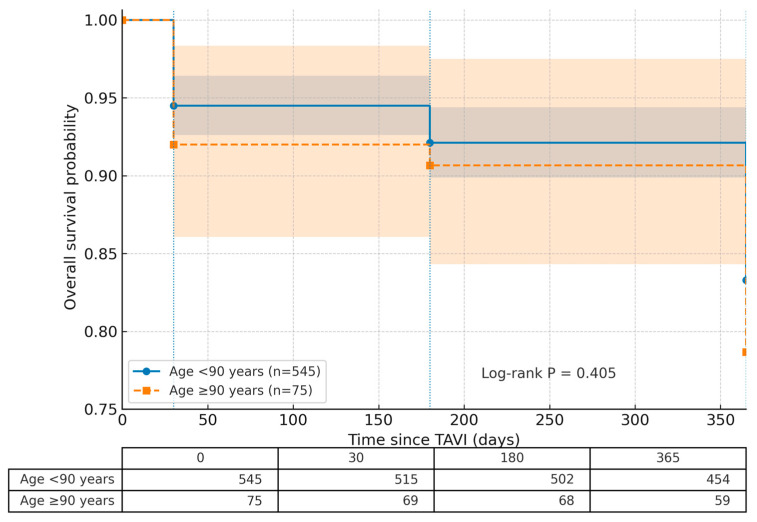
Kaplan–Meier analysis of all-cause mortality in patients aged <90 years and ≥90 years. Overall survival probability was not significantly different between the two age groups (≥90 years: 331.2 ± 11.7 days; 95% confidence interval [CI], 308.2–354.2; <90 years: 342.5 ± 5.6 days; 95% CI, 331.5–353.5; log-rank *p* = 0.405).

Finally, Table 5 displays the comparison of follow-up outcomes between the age groups. A significant difference was observed in NYHA classification at 1-month follow-up (*p* = 0.017), attributed to a lower proportion of NYHA Class 1 patients and a higher proportion of NYHA Class 2 and Class 3 patients in the ≥90 years group compared to the <90 years group. Additionally, although the 1-month, 6-month, and 1-year mortality rates were higher in the ≥90 years group than in the <90 years group, these differences were not statistically significant (*p* = 0.425, *p* = 0.838, *p* = 0.405, respectively). Figure 2 displays Kaplan–Meier curve demonstrating all-cause mortality over time for patients aged <90 years and ≥90 years.

## 8. Discussion

In this study evaluating the outcomes of TAVI in nonagenarians, 620 individuals were included, of whom 75 (12.1%) were aged 90 years or older. Patients aged ≥90 years had significantly lower BMI and lower prevalence of comorbidities such as DM, HL, and prior CABG. Additionally, they also had a more reduced baseline GFR. Echocardiographically, they exhibited higher baseline EF and septal thickness but lower LVEDD and LVESD, indicating a stiffer left ventricle in the older patient group. Procedural characteristics were largely similar between groups, apart from less frequent use of 29 mm valves in the ≥90 years group. While in-hospital stroke was more common among nonagenarians, other in-hospital outcomes did not differ significantly. Although NYHA class distribution at 1 month was less favorable in the ≥90 group, mortality rates at 1, 6, and 12 months were not significantly different between the groups.

We acknowledge that the retrospective nature of our study introduces the potential for selection bias. However, patient eligibility was determined through a structured Heart Team process, emphasizing clinical suitability rather than age alone. Nonagenarians in our cohort generally had fewer comorbidities, indicating careful selection of robust elderly patients. This may explain the similar mortality rates despite a higher stroke risk in this group. In our cohort, patients aged 90 years and older comprised 12.1% of the total TAVI population, reflecting a notable representation of nonagenarians in contemporary practice and aligning with the literature. This proportion is notably higher than that reported in the SWISS TAVI registry, where only 7.1% of patients were nonagenarians [2]. Likewise, Vendrik et al. reported a nonagenarian cohort comprising 8% of their study population [10]. In contrast, data from the France-2 and STS/ACC Transcatheter Valve Therapy Registry (TVT Registry) showed higher rates, with 15.4% and 15.7% of patients being 90 years or older, respectively [11,12]. Another study by Yokoyama et al. reported a nonagenarian population of 12.2%, similar to our cohort [13]. Similarly, Stehli et al. reported a comparable proportion of nonagenarians at 12.1%, which aligns closely with the findings of our study [14].

In our study population, the overall median STS score was 7.40 (IQR: 5.91–10.10), with comparable values observed in patients aged <90 years [6.70 (5.60–10.38)] and those aged ≥90 years [7.48 (5.98–10.00)], indicating no statistically significant difference in surgical risk between the two age groups. As demonstrated by baseline clinical profiles in previous studies, younger patients undergoing TAVI frequently present with a higher burden of comorbidities, including diabetes, hyperlipidemia, peripheral artery disease, chronic obstructive pulmonary disease, prior myocardial infarction, and previous cardiac surgeries [2,12]. In line with this, our study found that patients aged ≥90 years had significantly lower BMI and a lower prevalence of comorbidities such as diabetes, hyperlipidemia, and prior CABG compared to those under 90. These findings support the notion that nonagenarians undergoing TAVI represent a self-selected group with fewer comorbid conditions, consistent with the existing literature. While advanced age remains a key factor in heart team decision-making for TAVI, the presence of comorbidities and elevated surgical risk often play an even more critical role, particularly in younger candidates. Importantly, being younger does not automatically equate to lower surgical risk.

Our study revealed a significantly higher incidence of in-hospital stroke among nonagenarians (6.7%) compared to patients younger than 90 years (2.2%), emphasizing a notable periprocedural neurologic risk in this very elderly population. This increase is in line with prior evidence from large-scale studies such as the SWISS TAVI Registry, which demonstrated a clear age-related trend in cerebrovascular complications following TAVI [2]. Similarly, meta-analyses have reported an elevated odds ratio for stroke in nonagenarian patients, further supporting this association [15]. While the overall stroke incidence in real-world TAVR populations generally ranges between 2% and 3%, our findings indicate that age ≥ 90 years may independently predispose patients to higher neurologic risk [16]. Several pathophysiologic mechanisms likely converge: greater aortic arch and valve calcification with friable plaque increases embolic load during wire/catheter manipulation and device deployment; more tortuous iliofemoral and arch anatomy necessitates additional instrumentation; and age-related cerebral small-vessel disease may lower the threshold for clinically overt ischemia. Given this elevated risk, pre-procedurally, meticulous CT-based assessment of arch/leaflet calcium burden and tortuosity, selective carotid duplex in high-risk patients, and Heart-Team discussion of neurovascular risk can refine case selection. Additionally, implementation of enhanced procedural strategies—including the use of cerebral embolic protection devices—should be considered to improve neurologic outcomes in this high-risk group [17].

Although all-cause mortality rates—including in-hospital, 1-month, 6-month, and 1-year mortality—were numerically higher in the nonagenarian group compared to patients under 90 years old in our study, these differences did not reach statistical significance. This observation is consistent with several previous studies, although the literature on this topic remains heterogeneous. For instance, in the SWISS TAVI registry, 1-month and 1-year mortality rates among nonagenarians were reported as 6.7% and 19.7%, respectively, both of which were statistically higher compared to younger patients [2]. Similarly, a meta-analysis by Liu et al. reported a 1-month mortality rate of 6.2% in nonagenarians, with 1-year mortality ranging from 12.1% to 15.5%, again showing statistically significant differences [15]. Data from the STS/ACC TVT registry also support this trend, with in-hospital, 30-day, and 1-year mortality rates of 6.4%, 8.8%, and 24.8%, respectively—each significantly higher than in patients younger than 90 [12]. Conversely, other studies have not demonstrated such differences. For example, Yokoyama et al. found comparable 3-year mortality rates between nonagenarians and younger cohorts [13]. Similarly, Stehli et al. reported no significant difference in 1-year mortality, and Vendrik et al. observed no statistically significant differences in survival at 1-, 2-, 3-, 4-, and 5-year time points [14]. The FRANCE registry also reported 30-day, 6-month, and 1-year mortality rates of 11.3%, 20.1%, and 27.7% in nonagenarians, respectively; while these figures were numerically higher, the differences were not statistically significant [11]. One likely contributor to the mortality outcome is limited statistical power; with 75 nonagenarians and 16 deaths at 1 year, the study may not be sufficiently powered to detect modest absolute differences. Beyond sample size, selection effects likely attenuated between-group differences. As we show, patients ≥ 90 years had fewer comorbidities and similar STS risk compared with younger patients, reflecting a Heart-Team-driven, highly selective pathway that enrolls more robust nonagenarians; this “healthy nonagenarian” effect can dilute mortality contrasts despite advanced age. Taken together, these findings suggest that although age is associated with a trend toward higher mortality, careful patient selection and procedural expertise can result in acceptable outcomes even among patients aged 90 and older.

Although our study contributes valuable real-world data on TAVI outcomes in nonagenarians, it is important to acknowledge that the relatively small sample size of patients aged ≥90 years (*n* = 75) may have limited the statistical power to detect significant differences in key outcomes such as mortality. Larger-scale meta-analyses involving tens of thousands of patients have consistently reported significantly higher rates of mortality in this age group [15,18]. The absence of statistical significance in our study may, therefore, reflect sample size limitations rather than a true lack of clinical difference. Nevertheless, our findings still align with the broader literature in demonstrating consistent trends toward increased event rates in very elderly patients.

Frailty, comorbidity burden, and baseline functional status are important determinants of post-TAVI outcomes. In our cohort, these factors were incorporated qualitatively into Heart Team decision-making, and quantitatively, we report STS scores, detailed comorbidity profiles, and NYHA class at baseline; however, standardized frailty metrics (e.g., Clinical Frailty Scale, gait speed, grip strength) and broader QOL instruments were not uniformly available due to the retrospective design. These considerations likely contributed to residual confounding and should temper causal inference. In terms of functional status, as assessed by NYHA classification, patients aged <90 years showed slightly better baseline values, although the difference was not statistically significant. Both age groups demonstrated a marked improvement in NYHA class at the 1-month follow-up, reflecting symptomatic relief after TAVI. However, the younger cohort (<90 years) exhibited significantly better NYHA functional class at 1 month compared to nonagenarians. These findings align with previous studies suggesting that although nonagenarians benefit from TAVI, their functional recovery may be more gradual. For example, Thourani et al., in a review of the PARTNER (Placement of Aortic Transcatheter Valves) trial, observed that while quality of life (QOL) improved significantly within 30 days after TAVI, scores were initially lower in nonagenarians compared to younger patients. By 1 year, however, QOL levels became comparable between age groups [19]. Similar findings were noted by Arsalan et al. in the STS/ACC TVT registry, where KCCQ scores significantly improved by 30 days post-TAVI; however, nonagenarians had notably lower scores compared to younger patients at that time. By 1 year, though, quality of life scores were comparable between the age groups [12]. This suggests that nonagenarians may experience a delayed functional improvement, possibly due to age-related physiological limitations, but can still achieve meaningful recovery over time. Our findings similarly highlight the importance of allowing sufficient time for older patients to realize the full symptomatic benefits of TAVI.

## 9. Conclusions

In this study, nonagenarian patients undergoing TAVI represented a self-selected group with fewer comorbidities compared to their younger counterparts. Despite their advanced age, they exhibited comparable procedural characteristics and similar short- and long-term mortality rates, although these were numerically higher. Notably, the incidence of in-hospital stroke was significantly greater among nonagenarians. These findings suggest that while advanced age remains an important consideration, carefully selected nonagenarian patients can derive meaningful clinical benefit from TAVI, with outcomes that are largely comparable to younger cohorts.

## 10. Limitations

This study has several limitations that should be acknowledged. First, its retrospective design inherently introduces the possibility of selection bias and limits the ability to establish causal relationships. Second, functional status and quality of life (QOL) were assessed solely using NYHA classification, which, while widely used, may not fully capture the patient-reported outcomes or subtle changes in functional capacity. More comprehensive and validated QOL instruments, such as the Kansas City Cardiomyopathy Questionnaire (KCCQ), were not available in our dataset. Additionally, this study has a relatively small number of nonagenarian patients, which may reduce the ability to detect statistically significant differences in clinical outcomes. This limitation restricts the generalizability of our findings and should be considered when interpreting the results. We did not perform multivariable adjustment because the number of outcome events—particularly within the ≥90-year cohort (*n* = 75; 16 deaths at 1 year)—was insufficient to support a stable model without overfitting, per standard events-per-variable guidance. Accordingly, we prioritized transparent, prespecified age-group comparisons; robust adjusted analyses will require larger, preferably multicenter datasets with standardized frailty and QOL measures. Lastly, the study lacks long-term follow-up data on QOL beyond the first month after TAVI, which limits our understanding of the sustained functional benefits, particularly in the nonagenarian population.

## Figures and Tables

**Table 1:** Baseline Characteristics of Patients by Age Groups.

	General Population (*n* = 620)	Age < 90 (*n* = 545)	Age ≥ 90 (*n* = 75)	*p*-Value
Age (years)	79.0 (73.3–84.0)	78.0 (72.0–83.0)	92.0 (90.0–97.0)	n/a
Gender				0.622 ^1^
Male	281 (45.3%)	249 (45.7%)	32 (42.7%)	
Female	339 (54.7%)	296 (54.3%)	43 (57.3%)	
BMI (kg/m^2^)	24.2 (23.0–28.0)	24.2 (23.0–28.0)	23.3 (23.0–25.8)	0.021 ^2^
NYHA				0.266 ^1^
2	159 (25.6%)	143 (28.2%)	16 (21.3%)	
3	352 (56.8%)	312 (57.2%)	40 (53.3%)	
4	95 (15.3%)	79 (14.5%)	16 (21.3%)	
Pulmonary edema	14 (2.3%)	11 (2.0%)	3 (4.0%)	
STS score	7.40 (5.91–10.10)	6.70 (5.60–10.38)	7.48 (5.98–10.00)	0.421 ^2^
COPD				0.637 ^1^
None	324 (52.3%)	285 (52.3%)	39 (52.0%)	
Mild	35 (5.6%)	33 (6.1%)	2 (2.7%)	
Medium	177 (28.5%)	153 (28.1%)	24 (32.0%)	
Severe	84 (13.5%)	74 (13.6%)	10 (13.3%)	
CVA	36 (5.8%)	34 (6.2%)	2 (2.7%)	0.295 ^3^
PAD	48 (7.7%)	43 (7.9%)	5 (6.7%)	0.888 ^4^
DM	179 (28.9%)	166 (30.5%)	13 (17.3%)	0.027 ^4^
HT	512 (82.6%)	453 (83.1%)	59 (78.7%)	0.429 ^4^
HL	308 (49.7%)	280 (51.4%)	28 (37.3%)	0.023 ^1^
AF	149 (24.1%)	130 (23.9%)	19 (25.7%)	0.849 ^4^
CKD	23 (3.7%)	22 (4.0%)	1 (1.3%)	0.342 ^3^
CAD				0.097 ^1^
Normal	198 (32.0%)	172 (31.6%)	26 (35.1%)	
Non-obstructive	268 (43.4%)	244 (44.9%)	24 (32.4%)	
Obstructive	152 (24.6%)	128 (23.5%)	24 (32.4%)	
ACS history				>0.999 ^5^
None	603 (97.3%)	530 (97.2%)	73 (97.3%)	
NSTEMI	8 (1.3%)	7 (1.3%)	1 (1.3%)	
STEMI	8 (1.3%)	7 (1.3%)	1 (1.3%)	
USAP	1 (0.2%)	1 (0.2%)	0 (0.0%)	
PCI history	80 (12.9%)	69 (12.7%)	11 (14.9%)	0.734 ^4^
CABG history	135 (21.8%)	133 (24.4%)	2 (2.7%)	<0.001 ^4^
BAV	74 (12.2%)	71 (13.3%)	3 (4.2%)	0.042 ^4^
History of previous valve surgery	26 (4.2%)	24 (4.4%)	2 (2.7%)	0.758 ^3^
Anticoagulant/Antiplatelet *				
ASA	460 (75.8%)	495 (75.9%)	55 (75.3%)	>0.999 ^4^
Clopidogrel	26 (4.3%)	20 (3.8%)	6 (8.2%)	0.113 ^3^
Warfarine	122 (20.1%)	108 (20.2%)	14 (19.2%)	0.957 ^4^
NOAC	22 (3.6%)	19 (3.6%)	3 (4.1%)	0.740 ^3^
Basal GFR	64.3 (48.0–81.0)	65.0 (48.0–82.0)	58.0 (46.0–71.0)	0.018 ^2^
HbA1c	5.95 (5.50–6.52)	5.95 (5.53–6.58)	5.88 (5.31–6.39)	0.135 ^2^
Pre-op HBG	11.7 ± 1.9	11.7 ± 1.9	11.4 ± 1.6	0.135 ^6^

* Prior to the TAVI procedure. BMI: body mass index; NYHA: New York Heart Association; STS: Society of Thoracic Surgeons; CAD: coronary artery disease; CABG: coronary artery bypass graft; COPD: chronic obstructive pulmonary disease; CVA: cerebro-vascular accident; PAD: peripheric artery disease; DM: diabetes mellitus; HT: hypertension; HL: hyperlipidemia; AF: atrial fibrillation; CKD: chronic kidney disease; GFR: Glomerular filtration rate; ACS: Acute coronary syndrome; PCI: percutaneous coronary intervention; CABG: coronary artery bypass graft; BAV: bicuspid aortic valve; ASA: acetylsalicylic acid; NOAC: Novel oral anticoagulants; HBG: hemoglobin. Descriptive statistics for continuous numerical variables are presented as median (25th percentile–75th percentile) or mean ± standard deviation. ^1^ Pearson’s χ^2^ test, ^2^ Mann–Whitney U test, ^3^ Fisher’s exact test, ^4^ Continuity-corrected χ^2^ test, ^5^ Fisher–Freeman–Halton test, ^6^ Student’s *t*-test.

**Table 2:** Baseline Echocardiographic Findings of Patients by Age Groups.

	General Population (*n* = 620)	Age < 90 (*n* = 545)	Age ≥ 90 (*n* = 75)	*p*-Value
Basal EF	55.0 (45.0–65.0)	55.0 (45.0–65.0)	60.0 (50.0–65.0)	0.048 ^1^
Basal LVEDD	4.60 (4.30–5.10)	4.70 (4.30–5.10)	4.40 (4.10–4.80)	<0.001 ^1^
Basal LVESD	2.90 (2.50–3.50)	3.00 (2.60–3.60)	2.70 (2.30–3.10)	<0.001 ^1^
Basal septal wall thickness	1.40 (1.20–1.50)	1.40 (1.20–1.50)	1.50 (1.30–1.60)	0.012 ^1^
Basal posterior wall thickness	1.30 (1.20–1.40)	1.30 (1.20–1.40)	1.30 (1.20–1.40)	0.159 ^1^
Basal LA dimension	4.60 (4.30–5.00)	4.60 (4.30–5.00)	4.60 (4.30–5.00)	0.720 ^1^
AS group				0.314 ^2^
Normal	412 (67.0%)	368 (68.0%)	44 (59.5%)	
LFLG	41 (6.7%)	35 (6.5%)	6 (8.1%)	
Paradoxal LFLG	9 (1.5%)	7 (1.3%)	2 (2.7%)	
VSAS	153 (24.9%)	131 (24.2%)	22 (29.7%)	
Basal AVA	0.69 (0.54–0.80)	0.70 (0.54–0.80)	0.67 (0.53–0.80)	0.304 ^1^
Basal aortic mean gradient	47.0 (41.0–58.0)	46.0 (41.0–58.0)	49.0 (41.0–60.3)	0.490 ^1^
Bazal PASP	40.0 (35.0–55.0)	40.0 (32.3–55.0)	45.0 (35.0–60.0)	0.200 ^1^
Bazal MR				0.556 ^3^
None	10 (1.6%)	9 (1.7%)	1 (1.4%)	
Trivial	134 (21.8%)	121 (22.4%)	13 (17.6%)	
1	261 (42.5%)	232 (43.0%)	29 (39.2%)	
2	132 (21.5%)	114 (21.1%)	18 (24.3%)	
3	70 (11.4%)	59 (10.9%)	11 (14.9%)	
4	7 (1.1%)	5 (0.9%)	2 (2.7%)	

EF: Ejection fraction; LVEDD: Left ventricular end-diastolic dimension; LVESD: Left ventricular end-systolic dimension; LA: Left atrium; AS: Aortic stenosis; LFLG: Low flow low gradient; VSAS: Very severe aortic stenosis; AVA: Aortic valve area; PASP: Pulmonary artery systolic pressure; MR: Mitral regurgitation. Descriptive statistics for continuous numerical variables are presented as median (25th percentile–75th percentile) or mean ± standard deviation. ^1^ Pearson’s χ^2^ test, ^2^ Fisher’s exact test, ^3^ Continuity-corrected χ^2^ test.

**Table 3:** Procedural Characteristics of Patients by Age Groups.

	General Population (*n* = 620)	Age < 90 (*n* = 545)	Age ≥ 90 (*n* = 75)	*p*-Value
Transfemoral closure				0.896 ^1^
Prostar	206 (35.2%)	180 (35.0%)	26 (36.6%)	
Cut-down	12 (2.0%)	11 (2.1%)	1 (1.4%)	
Proglide	368 (62.8%)	324 (62.9%)	44 (62.0%)	
Pre-dilatation	435 (71.2%)	384 (71.4%)	51 (69.9%)	0.897 ^2^
Post-dilatation	19 (3.1%)	19 (3.5%)	0 (0.0%)	0.150 ^3^
Valve size				0.026 ^4^
20	3 (0.5%)	2 (0.4%)	1 (1.4%)	
23	263 (42.6%)	225 (41.4%)	38 (51.4%)	
25	16 (2.6%)	15 (2.8%)	1 (1.4%)	
26	252 (40.8%)	220 (40.4%)	32 (43.2%)	
27	7 (1.1%)	7 (1.3%)	0 (0.0%)	
29	77 (12.5%)	75 (13.8%) ^A^	2 (2.7%) ^A^	
Valve type				0.293 ^4^
Sapien XT	530 (85.8%)	470 (86.4%)	60 (81.1%)	
Edwards Sapien 3	50 (8.1%)	42 (7.7%)	8 (10.8%)	
Myval	29 (4.7%)	25 (4.6%)	4 (5.4%)	
Accurate neo	7 (1.1%)	6 (1.1%)	1 (1.4%)	
Evolut R	2 (0.3%)	1 (0.2%)	1 (1.4%)	
Valve in valve	9 (1.5%)	7 (1.3%)	2 (2.7%)	0.298 ^3^

^1^ Pearson’s χ^2^ test, ^2^ Continuity-corrected χ^2^ test, ^3^ Fisher’s exact test, ^4^ Fisher–Freeman–Halton test. ^A^ The difference between the groups is statistically significant (*p* = 0.012).

**Table 4:** In-Hospital Outcomes of Patients by Age Groups.

	General Population (*n* = 620)	Age <90 (*n* = 545)	Age ≥ 90 (*n* = 75)	*p*-Value
Stroke	17 (2.7%)	12 (2.2%)	5 (6.7%)	0.044 ^1^
Pericardial Tamponade				0.715 ^2^
None	607 (97.9%)	534 (98.0%)	73 (97.3%)	
Yes	11 (1.8%)	9 (1.7%)	2 (2.7%)	
Percutaneous Surgical drainage needed	2 (0.3%)	2 (0.4%)	0 (0.0%)	
Post-op Arrhythmias				0.774 ^2^
None	536 (86.7%)	471 (86.6%)	65 (87.8%)	
Complete AV block	44 (7.1%)	37 (6.8%)	7 (9.5%)	
AF	21 (3.4%)	20 (3.7%)	1 (1.4%)	
LBBB	14 (2.3%)	13 (2.4%)	1 (1.4%)	
VT	3 (0.5%)	3 (0.6%)	0 (0.0%)	
Other Complications				0.477 ^2^
None	593 (95.6%)	522 (95.8%)	71 (94.7%)	
Closure device fail	14 (2.3%)	11 (2.0%)	3 (4.0%)	
Valve embolization	4 (0.6%)	4 (0.7%)	0 (0.0%)	
AKI	4 (0.6%)	4 (0.7%)	0 (0.0%)	
Coronary obstruction	2 (0.3%)	2 (0.4%)	0 (0.0%)	
Valve in valve	2 (0.3%)	1 (0.2%)	1 (1.3%)	
Annular rupture	1 (0.2%)	1 (0.2%)	0 (0.0%)	
Peripheral Complication				0.542 ^2^
None	576 (92.9%)	507 (93.0%)	69 (92.0%)	
Hematoma	9 (1.5%)	7 (1.3%)	2 (2.7%)	
Pseudoaneurysm	10 (1.6%)	9 (1.7%)	1 (1.3%)	
Dissection	21 (3.4%)	19 (3.5%)	2 (2.7%)	
Major bleeding	4 (0.6%)	3 (0.6%)	1 (1.3%)	
Post-op Pacemaker				0.833 ^2^
None	572 (92.6%)	504 (92.6%)	68 (91.9%)	
Permanent	45 (7.3%)	39 (7.2%)	6 (8.1%)	
Temporary	1 (0.2%)	1 (0.2%)	0 (0.0%)	
Procedural Success	595 (96.1%)	522 (96.0%)	73 (97.3%)	0.756 ^1^
Hospital stay (day)	4.0 (3.0–5.0)	4.0 (3.0–5.0)	4.0 (3.0–5.0)	0.329
In-hospital mortality	26 (4.2%)	20 (3.7%)	6 (8.0%)	0.114 ^1^

AV: Atrioventricular; AF: Atrial fibrillation; LBBB: Left bundle branch block; VT: Ventricular tachycardia; AKI: Acute kidney injury. ^1^ Fisher’s exact test, ^2^ Fisher–Freeman–Halton test.

**Table 5:** Follow-Up Outcomes of Patients by Age Groups.

	General Population (*n* = 620)	Age < 90 (*n* = 545)	Age ≥ 90 (*n* = 75)	*p*-Value
1-month NYHA				0.017 ^1^
1	178 (30.5%)	167 (32.4%)	11 (15.9%)	
2	372 (63.7%)	320 (62.1%)	52 (75.4%)	
3	34 (5.8%)	28 (5.4%)	6 (8.7%)	
1-month mortality	36 (5.8%)	30 (5.5%)	6 (8.0%)	0.425 ^2^
6-month mortality	50 (8.1%)	43 (7.9%)	7 (9.3%)	0.838 ^3^
1-year mortality	107 (17.3%)	91 (16.7%)	16 (21.3%)	0.405 ^3^

NYHA: New York Heart Association. ^1^ Pearson’s χ^2^ test, ^2^ Fisher’s exact test, ^3^ Continuity-corrected χ^2^ test.

## Data Availability

The datasets used and/or analyzed during the current study are available from the corresponding author on reasonable request.
